# Analysis of Structural and Functional Differences of Glucans Produced by the Natively Released Dextransucrase of *Liquorilactobacillus hordei* TMW 1.1822

**DOI:** 10.1007/s12010-020-03407-6

**Published:** 2020-08-21

**Authors:** Jonas Schmid, Daniel Wefers, Rudi F. Vogel, Frank Jakob

**Affiliations:** 1 Chair of Technical Microbiology, Technical University of Munich (TUM), Freising, Germany; 2 Division of Food Chemistry, Institute of Chemistry, Martin-Luther-University Halle-Wittenberg, Halle (Saale), Germany; 3 Department of Food Chemistry and Phytochemistry, Karlsruhe Institute of Technology, Karlsruhe, Germany

**Keywords:** Lactic acid bacteria, Dextransucrase, Dextran, Molecular weight, Rheology, Panose

## Abstract

The properties of the glucopolymer dextran are versatile and linked to its molecular size, structure, branching, and secondary structure. However, suited strategies to control and exploit the variable structures of dextrans are scarce. The aim of this study was to delineate structural and functional differences of dextrans, which were produced in buffers at different conditions using the native dextransucrase released by *Liquorilactobacillus* (L.) *hordei* TMW 1.1822. Rheological measurements revealed that dextran produced at pH 4.0 (*M*_*W*_ = 1.1 * 10^8^ Da) exhibited the properties of a viscoelastic fluid up to concentrations of 10% (w/v). By contrast, dextran produced at pH 5.5 (*M*_*W*_ = 1.86 * 10^8^ Da) was gel-forming already at 7.5% (w/v). As both dextrans exhibited comparable molecular structures, the molecular weight primarily influenced their rheological properties. The addition of maltose to the production assays caused the formation of the trisaccharide panose instead of dextran. Moreover, pre-cultures of *L. hordei* TMW 1.1822 grown without sucrose were substantial for recovery of higher dextran yields, since the cells stored the constitutively expressed dextransucrase intracellularly, until sucrose became available. These findings can be exploited for the controlled recovery of functionally diverse dextrans and oligosaccharides by the use of one dextransucrase type.

## Introduction

The utilization of microbial exopolysaccharides (EPSs) is of growing importance and commercial interest due to their great structural diversity and concomitant manifold properties. They can be used instead of synthetically produced additives in cosmetics and food products and may replace commonly used emulsifiers because of their water-binding capacity or rather hydrocolloid properties [1–5]. Furthermore, EPSs may play a future role in applications like tissue engineering, drug delivery systems, and other medical applications [6–10] or even fields like biofuel research [11], as they are usually involved in the formation of biofilms [12–19].

One of the most prominent EPSs is dextran, which is commercially exploited in fermented foods like sourdoughs, panettone, or fruit juices, and applied as blood plasma volume expander or as stationary phase of size-exclusion columns [6, 20–24]. It is composed of α-(1,6)-linked glucose monomers (backbone), which can be branched at positions *O*2, *O*3, or *O*4 depending on the catalytic domain of their producing dextransucrases (EC 2.4.1.5) [25]. These extracellular enzymes are exclusively expressed by lactic acid bacteria and belong to the family 70 of glycoside hydrolases according to the CAZy database. They use the energy of the glycosidic bond of sucrose for polymerization of the contained glucose moiety while fructose is continuously released [20, 26–30]. The size, structure, and yield of sucrose-derived EPSs like dextrans are multiply influenced by extrinsic factors, e.g., pH, temperature, or substrate concentration [31–38], but also by the genetic constitution and physiology of the bacterial producer strain [25, 39]. Moreover, if present, maltose can be involved in the dextransucrase (acceptor) reaction. This usually results in the formation of diverse short-chain gluco-oligosaccharides that exhibit a low degree of polymerization and are promising prebiotics [30, 40–43]. The extreme differences in possible chain lengths contribute to the variable functional properties of the produced dextrans and other sucrase-derived EPSs [33, 35, 44, 45] and can be controlled during production for the manufacture of tailor-made EPSs exhibiting the desired properties. By contrast, the structures and sizes of other commercially used plant-derived hydrocolloids such as cellulose or starch are comparably fixed, why they need to be synthetically modified to obtain varying hydrocolloid properties [46–49]. In a previous study, we were able to produce high molecular weight dextrans of different sizes via the application of different pH values upon the polymerization process using the dextransucrase natively released by *Liquorilactobacillus hordei* TMW 1.1822. As these dextrans appeared to differ regarding their thickening properties depending on their molecular size [50], we wanted to elucidate their basic structural and rheological differences. In this way, we wanted to get novel insights into the structure–function relationship of these dextrans, which are essential for their knowledge-based application as hydrocolloids. Moreover, the impact of maltose on dextran formation should be elucidated for possible further extension and exploitation of the product spectrum of this dextransucrase.

## Material and Methods

### Strain, Standard Media and Basic Growth Kinetics

*Liquorilactobacillus hordei* TMW 1.1822 was originally isolated from water kefir [51] and cultivated at 30 °C on modified MRS (mMRS) as described before [50, 52], containing 10 g/L peptone from casein, 5 g/L yeast extract, 5 g/L meat extract, 4 g/L K_2_HPO_4_, 2.6 g/L KH_2_HPO_4_, 3 g/L NH_4_Cl, 1 g/L Tween 80, 0.5 g/L cysteine-HCl, 0.2 g/L MgSO_4_·7H_2_O, 0.036 g/L MnSO_4_·H_2_O, and 15 g/L agar for solid media, and a pH of 6.2. For the determination of the growth kinetics depending on different sugar sources, TMW 1.1822 was cultivated in mMRS containing either glucose + fructose (each 10 g/L) or sucrose (19 g/L) for 24 h. In these experiments, the pH, colony-forming units (CFUs), and dextran yields were determined after 0, 4, 8, 14, 19, and 24 h. For verification of the strain identity and to exclude any contamination, matrix-assisted laser desorption–ionization time-of-flight mass spectrometry (MALDI-TOF-MS) was randomly performed and validated through our in-house database [53].

### Production of High Molecular Weight Dextrans Exhibiting Different Sizes

Dextran production was performed according to Schmid et al. [50] and is briefly described. The mMRS broths (glucose + fructose each 10 g/L) were inoculated with 4% of 20-h pre-cultures with an adjusted OD_590nm_ of 2.0, to obtain cells in the exponential growth phase after 18 h at 30 °C. The media supernatants were removed after centrifugation (10000×*g*, 10 min), followed by the addition of 0.1 M citrate-phosphate buffer containing 0.1 M sucrose with pH 4.0 or 5.5. After 3 h of static incubation at 30 °C, the cells were removed by centrifugation (10000×*g*, 10 min) and filtration (0.2-μm nylon filters, Phenomenex, Germany). Subsequently, dextran production with the dextransucrase containing supernatants in the respective buffers (pH 4.0 and 5.5) was performed for 24 h at 30 °C. Additionally, one intermediate condition was analyzed, where the cells were not removed, and therefore, uncontrolled pH decrease occurred due to the metabolic activity of the remaining cells (UC).

### Dextran Formation After Pre-Cultivation in mMRS Broths Media Containing Sucrose

To study the influence of sucrose in pre-cultivation on the dextran formation (3.3; Fig. 3), the sugar compositions in mMRS were varied. Therefore, mMRS was either supplemented with 10 g/L glucose + 10 g/L fructose (glc + fru), 5 g/L glucose + 5 g/L fructose + 9.5 g/L sucrose (glc + fru + suc), or 19 g/L sucrose (suc). The respective sugar amounts were adjusted to obtain almost equal total sugar molarities (~ 0.05 M), respectively. These media were also inoculated with 4% of 20-h pre-cultures grown in mMRS (glc + fru) with an adjusted OD_590nm_ of 2.0, to obtain cells in the exponential growth phase after 18 h. The cells were then incubated in a 0.1 M citrate-phosphate buffer pH 4.5 [50] containing 0.1 M sucrose, yielding native dextransucrase for subsequent enzymatic dextran formation, as stated in the “Production of High Molecular Weight Dextrans Exhibiting Different Sizes” section.

### Dextran Production in the Presence of Maltose and Sucrose

For the investigation of the influence of maltose on the products of the dextransucrase, the experimental setup depicted in Fig. 3 was carried out. For this purpose, we used the cells from the three different pre-cultivations, which are described in the “Dextran Formation After Pre-Cultivation in mMRS Broths Media Containing Sucrose” section. As described in the “Production of High Molecular Weight Dextrans Exhibiting Different Sizes” section, the supernatant was removed, and a 0.1 M citrate-phosphate buffer pH 4.5 [50] containing 0.1 M sucrose + 0.1 M maltose was added, followed by the dextran production for 24 h at 30 °C.

### Dextran Production Using Dextransucrase Containing Cell Lysates

Additionally, cells obtained from mMRS with glucose + fructose (each 10 g/L) or sole sucrose (19 g/L) were recovered after 18 h of incubation by centrifugation and lysed with 10 μg/mL lysozyme at 37 °C overnight. The lysates were subsequently re-dissolved in 0.1 M citrate-phosphate buffer pH 4.5 containing 0.1 M sucrose and incubated for 24 h at 30 °C.

### Dextran Isolation

Dextran isolation was performed as previously described [54] by applying an initial centrifugation step (10000×*g*, 30 mins) for removal of insoluble material, precipitation of water-soluble dextrans with 2 volumes of ethanol for 24 h at 4 °C, dialysis against ddH_2_O at 4 °C for 48 h (3500-Da cutoff; SERVAPOR, SERVA Electrophoresis GmbH, Germany), and applying a final lyophilization step.

### Analysis of the Macromolecular Structure and Size of Dextrans by AF4-MALS-UV

To determine the molecular weights and the root mean square (rms) radii of the isolated dextrans, asymmetric flow field-flow fractionation (AF4) coupled with multi-angle laser light scattering (MALS) (Dawn Heleos II, Wyatt Technologies, Germany) analysis and UV detection (Dionex Ultimate 3000, Thermo Scientific, USA) was performed. The lyophilized dextrans were re-dissolved in 0.05 M NaNO_3_, which furthermore served as the eluent. A total of 100 μL of the respective samples with a concentration of 0.1 mg/mL was injected and separated in the long channel (LC) using a 10-kDa regenerated cellulose membrane, a detector flow rate of 1 mL/min, and a cross-flow gradient of 3 to 0.1 mL/min over 15 min. Subsequently, the cross-flow was kept constant at 0.1 mL/min for 15 min. The dextrans produced from sucrose at different pH [50] were characterized regarding their molecular size and structure. The MALS analysis was performed with all 17 detectors, and the chromatograms were further analyzed and evaluated with the ASTRA 6.1 software (Wyatt Technologies, Germany). The model berry 1 was most suited for evaluation of the dextrans (*R*^2^ > 0.90), whereby the rms radii were determined in the particle mode. In advance of the molecular mass calculation, the extinction coefficients of the dextrans were determined in 96-well microtiter plates at *λ* = 400 nm using concentrations of 0.5–2.5 mg/mL (SPECTROstar, BMG LABTECH) according to the Beer–Lambert law. Finally, the extinction coefficients were used for mass calculations using UV-based concentration detection of the separated dextran fractions at *λ* = 400 nm (Dionex Ultimate 3000, Thermo Scientific, USA).

### Analysis of the Glycosidic Linkages of Dextrans by Methylation Analysis

Methylation analysis was carried out as described previously by Fels et al. [18]. Briefly, dextrans were methylated in DMSO by using powdered sodium hydroxide and methyl iodide. Methylated polysaccharides were extracted by using dichloromethane, dried and hydrolyzed by using 2 M trifluoroacetic acid (90 min, 121 °C). After evaporation of the acid, the partially methylated monosaccharides were reduced by using sodium borodeuteride. The reaction was terminated by using glacial acetic acid, and 1-methylimidazole and acetic anhydride were added for acetylation. The obtained partially methylated alditol acetates were identified by using GC-MS and semiquantitatively analyzed by using GC-FID with the previously described conditions (molar response factors according to Sweet et al. [55]). All analyses were performed in duplicate.

### Characterization of the Rheological Properties of High Molecular Weight Dextrans

For determination of the basic rheological properties of the produced dextrans, flow curves were recorded and the capability of gel formation by the respective dextrans was studied using the rheometer Physica MCR 501 (Anton Paar, Austria). For the measurements of samples with lower viscosity (< 7.5% w/v), the concentric cylinder geometry CC 27-SS was used; for samples of higher viscosity, the cone–plate geometry CP 25-1 was used (Anton Paar, Austria). To re-dissolve the dextran in ddH_2_O, the samples were vortexed for 10 min, heated in a water bath at 55 °C for 45 min, and centrifuged (10000×*g*, 10 min) for removal of air bubbles. The measurements were performed at 20 °C and a shear rate ranging from 0.1 to 1000 s^-1^. To determine the linear viscoelastic (LVE) region, strain sweep tests were performed at 1 rad/s with stress ranging from 0.1 to 100%, followed by frequency sweep tests at angular frequencies of 0.1 to 100 rad/s and 1.0% strain. Concentrations of highest differences and interest were picked for visualization, resulting in 7.5, 5, 2.5, and 1% w/v for the rotational tests (concentric cylinder) and 7.5, 10, and 12.5% w/v for the strain/frequency sweep tests (cone–plate).

### Detection of Mono- and Disaccharides by HPLC-RI

Mono- and disaccharides were quantified by high-performance liquid chromatography (HPLC) coupled to refractive index detection (ERC Refractomax 521, Thermo Fisher Scientific, USA). Sugar separations were performed on a Rezex RPM-Monosaccharide Pb^2+^ column (Phenomenex Ltd., Germany) with water as mobile phase at a constant flow rate of 0.6 mL/min at 85 °C and 20 μL of injection volume. For identification and quantification of the respective sugars, sugar standards were used and the calibration curves were generated with the Chromeleon^TM^ software (version 6.8, Dionex, Germany).

### Identification of Low Molecular Weight Oligosaccharides by HPAEC-PAD

For identification of short-chain gluco-oligosaccharides produced by dextransucrases in the presence of maltose (“Dextran Formation After Pre-Cultivation in mMRS Broths Media Containing Sucrose”), high-performance anion-exchange chromatography (HPAEC) with pulsed amperometric detection (PAD) (ICS5000, Thermo Fisher Scientific, USA) on a CarboPac PA20 column (Thermo Fisher Scientific, USA) was performed. The separation was accomplished at a flow rate of 0.5 mL/min and an isocratic elution with 150 mM NaOH (Merck Millipore, USA) for 80 min and final flushing step with 200 mM NaOH and 1 M sodium acetate (Merck Millipore, USA) for 20 min. For identification and quantification of oligosaccharides, external sugar standards were used (Carbosynth, Switzerland) and the calibration curves were generated with the Chromeleon^TM^ software (version 6.8, Dionex, Germany).

## Results

### Molecular Size and Structure of High Molecular Weight Dextrans

The dextrans produced in buffer at pH 5.5 were of bigger size (Table 1, 94.3 ± 0.95 nm) and higher molecular weight (1.86 ± 0.02 × 10^8^ Da) compared with dextrans produced at pH 4.0 (73.5 ± 0.7 nm and 1.09 ± 0.04 × 10^8^) and the dextran UC produced at uncontrolled pH (77.3 ± 1.0 nm and 1.40 ± 0.02 × 10^8^ Da). The polydispersity, which was calculated by *M*_*W*_/*M*_*n*_ and *R*_*W*_/*R*_*n*_, was similar among the samples (Table 1). The hydrodynamic coefficients *v*_*g*_, which are obtained by double-logarithmic plotting of rms radii vs. molar masses over the entire polymer fraction [34], were in a comparable range of 0.74–0.79 (Table 1), indicating similar spatial (secondary) structures of the dextran molecules in aqueous solution as reported previously [5]. The molecular structure of the samples was further investigated by methylation analysis (Table 2) and confirmed the presence of dextrans with a 1,6-linked backbone (1,6-linked glucose), which is branched at position *O*3 (1,3,6-linked glucose) to a low extent. The portions of the glycosidic linkages only showed slight variations among the samples, which suggests a comparable molecular structure for the investigated polysaccharides.

**Table 1:** Molecular weights, radii, distribution quotient, and slope of confirmation plot determined by AF4-MALS-UV for dextrans produced at pH 5.5, 4.0, and uncontrolled (UC)

	*M*_*w*_ (Da)	*M*_*w*_/*M*_*n*_	*R*_*w*_ (nm)	*R*_*w*_/*R*_*n*_	Slope of confirmation plot
pH 4.0	1.09 ± 0.04 × 10^8^	1.10 ± 0.01	73.5 ± 0.7	1.13 ± 0.02	0.74 ± 0.1
pH 5.5	1.86 ± 0.02 × 10^8^	1.09 ± 0.01	94.3 ± 0.9	1.11 ± 0.01	0.77 ± 0.2
UC	1.40 ± 0.02 × 10^8^	1.08 ± 0.00	77.3 ± 1.0	1.12 ± 0.01	0.79 ± 0.2

**Table 2:** Glycosidic linkage (mol %) of dextrans produced at pH 5.5, pH 4.0, and uncontrolled (UC) pH as determined by methylation analysis

Glycosidic linkage	pH 5.5	UC	pH 4.0
t-Glc*p*	5.0 ± 0.0	4.9 ± 0.0	5.0 ± 0.2
1,3-Glc*p*	1.4 ± 0.0	1.5 ± 0.0	1.5 ± 0.0
1,6-Glc*p*	90.9 ± 0.0	90.6 ± 0.0	90.8 ± 0.3
1,3,6-Glc*p*	2.8 ± 0.1	3.0 ± 0.1	2.6 ± 0.1

### Rheological Properties of the High Molecular Weight Dextrans

**Fig. 1:**
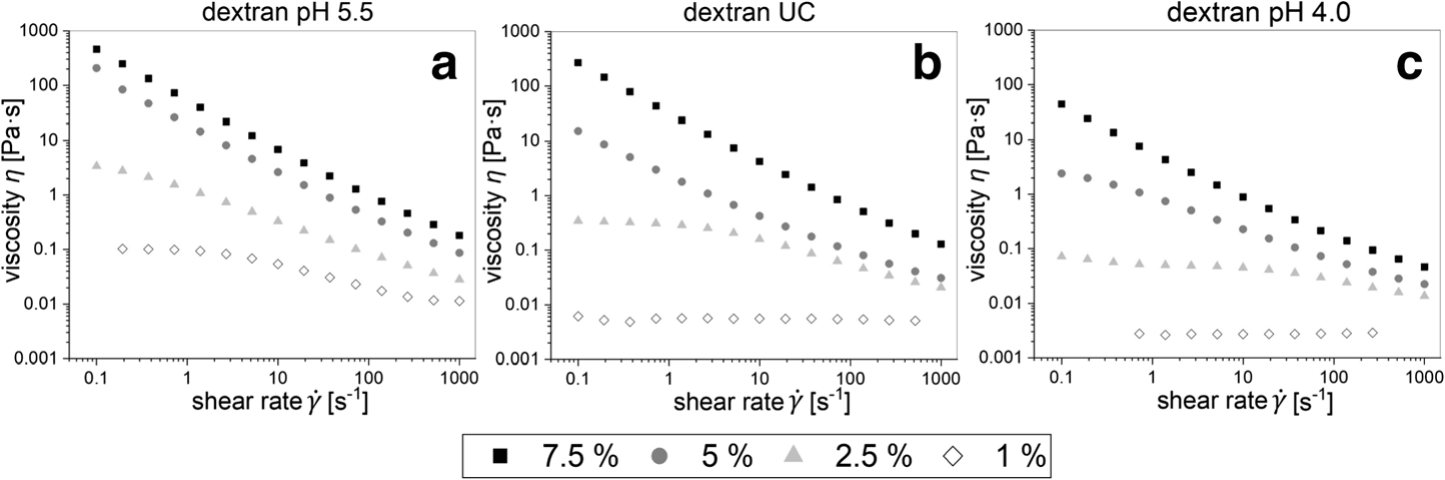
Concentration-dependent viscosity curves of dextrans produced at pH 5.5 (A), uncontrolled pH (UC) (B), and pH 4.0 (C)

The high molecular weight dextrans described in the “Molecular Size and Structure of High Molecular Weight Dextrans” section were further analyzed regarding their flow and viscoelastic behavior. Figure 1 depicts the flow curves of the dextrans produced at pH 4.0 and 5.5 and of the dextran produced without pH control (UC). The dextran pH 5.5 showed shear thickening behavior for all tested specific concentrations 1, 2.5, 5, and 7.5% (w/v) (Fig. 1A) and the highest detected viscosity of around 460 Pa/s (shear rate 0.1 s^-1^). In contrast, dextran pH 4.0 (Fig. 1C) exhibited the comparatively lowest viscosities at equally applied specific concentrations and no distinct shear thinning behavior at concentrations below 5% (w/v), while the dextran UC (Fig. 1B) was in between dextrans pH 4.0 and 5.5 regarding determined viscosities at equal test conditions.

For the determination of the upper limits of the LVE region, amplitude sweep tests were performed. At concentrations of 12.5% (w/v), all samples showed a gel-like behavior with G″ (loss modulus) < G′ (storage modulus). The highest storage and loss moduli were measured for dextran pH 5.5 followed by dextran UC and dextran pH 4.0 (Fig. 1A). The loss of the gel-like behavior for dextrans pH 4.0 at a concentration of 10% w/v was evident due to no crossover of G′ and G″. A similar observation was made for dextran UC at a specific concentration of 7.5% (w/v) (Fig. 1B and C). Dextrans pH 4.0 could not be analyzed at 7.5% (w/v) due to its distinct fluid-like behavior.

**Fig. 2:**
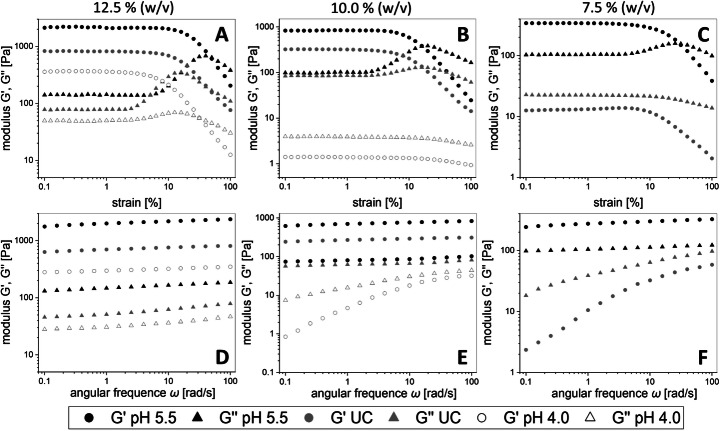
Strain sweep (A–C) and frequency sweep (D–F) tests depicting storage (G′, circle) and loss (G″, triangle) moduli of dextrans produced at pH 5.5 (black), uncontrolled pH (UC, gray), and pH 4.0 (white). The measurements were performed by applying the specific concentrations 12.5% (A, D), 10% (B, E), and 7.5% (w/v) (C, F) of each dextran, respectively

In Fig. 2, the results of the frequency sweep tests performed at a constant strain of 1.0% are depicted. The highest shear storage moduli G′ and loss moduli G″ were determined for dextran pH 5.5, while G′ > G″ was measured at each concentration for this dextran in contrast to dextrans UC (Fig. 2F) and pH 4.0 (Fig. 2E).

### Influence of Sucrose in the MRS Pre-Cultivation Medium on the Dextran Production in Buffers

**Fig. 3:**
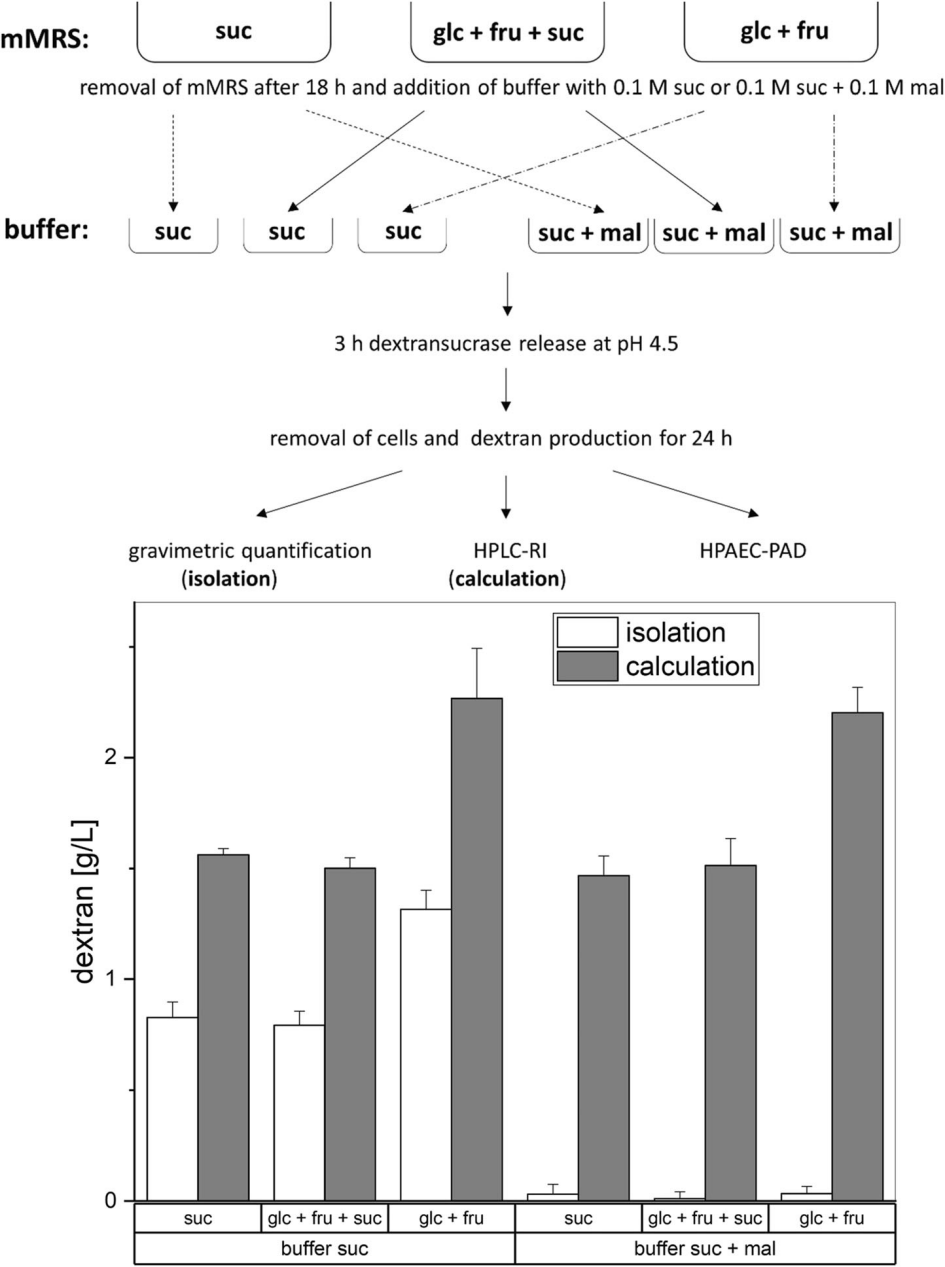
Top: overview of the experimental setups used to characterize extracellular dextran production depending on the presence of sucrose in the pre-cultivation and maltose during dextran formation. Cells were cultivated in medium containing solely sucrose (suc), glucose + fructose + sucrose (glc + fru + suc), and glucose + fructose (glc + fru) for 18 h; harvested; and re-dissolved in 0.1 M citrate-phosphate buffer pH 4.5. This buffer was supplemented with either 0.1 M sucrose (suc) or 0.1 M sucrose + 0.1 M maltose (suc + mal). After 3 h of incubation, the cells were removed by centrifugation and sterile filtration. The cell-free supernatants were incubated for 24 h, followed by dextran isolation, and HPLC-RI and HPAEC-PAD measurements. Bottom: dextran yields for the described experiment. The bars indicate the produced dextran amounts in g/L, which were determined either gravimetrically (isolation) or via the calculated transglycosylation activity and the resulting dextran amounts after 24 h (calculation). Data are expressed with mean ± SD of three biological replicates

**Fig. 4:**
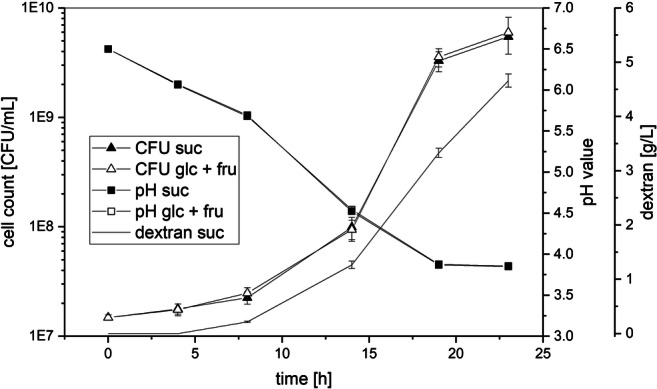
Monitoring of the colony-forming units (CFU), pH, and dextran formation of *L. hordei* TMW 1.1822 during cultivation in either mMRS medium containing glucose + fructose (glc + fru) or mMRS medium containing solely sucrose (suc) as respective carbohydrate sources. Dextran yields are only shown for mMRS with sucrose since no dextran formation took place without sucrose

The method to produce dextran under controlled pH (“Production of High Molecular Weight Dextrans Exhibiting Different Sizes”) [50] was slightly modified to study the influence of sucrose in the pre-cultivation medium on the produced dextran amounts in buffers (Fig. 3, “Dextran Formation After Pre-Cultivation in mMRS Broths Media Containing Sucrose”). The amounts of produced dextrans were determined by weighing after isolation and by calculation of the totally produced dextran amounts using the equation c (transglycosylated glucose) × 162.16 g/mol (molar mass of glucose in dextran). If sucrose was present in the mMRS pre-cultivation medium, the dextran yields obtained after production in buffers were considerably lower compared with those obtained without sucrose (Fig. 3, bottom). Furthermore, no significant differences in the produced dextran amounts were observed, if *L. hordei* TMW 1.1822 had been pre-cultivated in mMRS medium with sucrose as the sole sugar source or with glucose/fructose/sucrose as mixed sugar sources. Similar results were obtained, if lysates of *L. hordei* TMW 1.1822 harvested from mMRS ± sucrose were used for dextran production (− suc: 1.29 ± 0.014 g/L; + suc: 0.71 ± 0.056 g/L). The growth and acidification of *L. hordei* TMW 1.1822 in mMRS with either glucose/fructose or sucrose as the carbon source were highly similar within 24 h of cultivation (Fig. 4). In the presence of sucrose, up to 4.5 g/L dextran was additionally produced.

### Impact of Maltose on the Product Specificity of the Dextransucrase

**Fig. 5:**
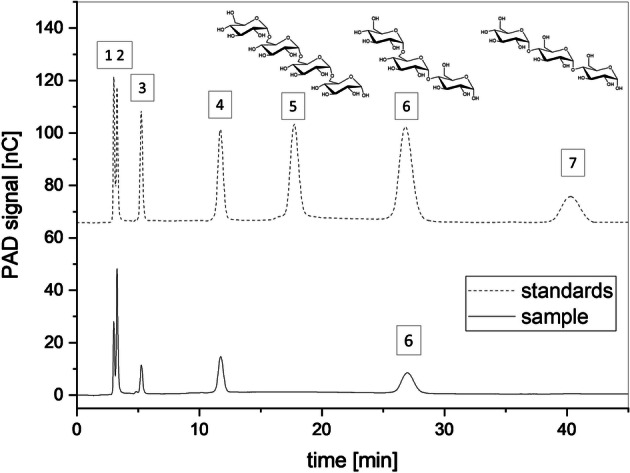
HPAEC-PAD chromatograms of the diluted supernatant from the production buffer containing sucrose + maltose and a standard solution containing the sugars glucose (1), fructose (2), sucrose (3), maltose (4), isomaltotetraose (5), panose (6), and maltotriose (7)

To study the impact of maltose on the product specificity of the dextransucrase released by *L. hordei* TMW 1.1822, maltose (0.1 M) was added to the production buffer containing 0.1 M sucrose. The amounts of isolated dextran were drastically reduced in the presence of maltose to less than 0.05 g/L (Fig. 3, right part). However, the overall activity of the dextransucrase was in a similar range as compared with the corresponding experimental series performed without maltose (Fig. 3, left part). As the concentrations of free glucose and fructose were similar among the particularly comparable approaches (± maltose), the previously reported initial hydrolysis of sucrose prior to transglycosylation by these dextransucrases [50] occurred at all tested conditions. The low molecular weight oligosaccharides formed in the presence of maltose were analyzed by using HPAEC-PAD (Fig. 5). Besides the peaks corresponding to the sugars added to the production buffers (glucose 1, fructose 2, sucrose 3, maltose 4), an additional peak [6] was detected. As determined by the retention time, this peak corresponds to panose [6] in the standard mixture, eluting after isomaltotetraose [5] and before maltotriose [7]. The determined average concentration of panose was 15 mM, which is consistent with the maltose reduction after 24 h (16 mM) and the calculated glucose used for transglycosylation (15 mM). Peaks at later retention times than 80 min, which might refer to longer glucose oligosaccharides, were not detected.

## Discussion

Dextransucrases are widely abundant among lactic acid bacteria (LAB), while their primary and secondary structures are highly variable [25, 27, 56]. Consequently, dextrans differing in molecular size, structure, branching, and secondary structure can be produced using different dextransucrases [56–59]. However, variations in the size and molecular weight as well as the molecular structure are also influenceable by extracellular factors [36–38, 60]. As we showed in our previous study [50], the size of dextran from *L. hordei* TMW 1.1822 is strongly dependent on the extracellular pH value during the dextran polymerization process. These dextrans empirically showed distinct differences in their gelling properties at equally applied concentrations [50]. Thus, one aim of the present study was to investigate these differences in more detail using rheological measurements. As reported in previous studies about microbial levans [37, 45], the viscosity of these dextrans increased with rising molecular size despite comparable molecular structure and degree of branching (Table 2). Comparable thickening and gelling properties were reported for dextrans, which were linear and had a higher molecular weight than the dextrans investigated in the present study [58]. The AF4-MALS-UV analysis revealed only minor differences in their polydispersity and random coil–like secondary structure in aqueous solution (Table 1) [5]. These findings indicate that the chain length/molecular weight is the most decisive factor influencing the variations in the rheological behavior of this dextran and other uncharged, water-soluble EPSs [37, 45]. Longer dextran molecules applied at critical concentrations may have more contact points for establishment of more intermolecular hydrogen bonds and of a more stable physical network than shorter dextran molecules [45]. Consequently, EPS production needs to be optimized towards the desired molecule size in addition to the final EPS yield. As EPS-producing LAB are frequently used as starter cultures for improvement of the structural properties of foods [61, 62], a proper pH control during food fermentation may allow the controlled in situ production of, e.g., gelling or thickening dextrans. This approach could replace the use of synthetically modified thickeners and gelling agents, which have to be labeled on food packages in contrast to EPSs produced naturally from sucrose by food-grade LAB [47, 61, 63–65]. Moreover, the portfolio of commercially available dextrans recovered from *Leuconostoc mesenteroides* B-512F [23, 58] could be extended by further triggered glucans that exhibit other molecular structures, sizes, and concomitant properties.

The influence of other extrinsic factors besides the pH value on the dextran polymerization, such as the availability of sugars and the applied sugar concentrations as well as the temperature, was subject of several studies [30, 42, 43, 66, 67]. The presented approach for dextran production revealed that the concentration of dextran was reduced if the bacterium was in contact with sucrose prior to the dextran polymerization process (Fig. 3). Hence, the intracellular dextransucrase reservoir of *L. hordei* TMW 1.1822 [25, 50] appeared to be partially dissipated, although its growth with or without sucrose was comparable (Fig. 4). This finding was confirmed by determination of the amounts of dextran produced in buffer using the crude cell lysates of *L. hordei* recovered with or without sucrose in the mMRS pre-cultivation medium, respectively (3.3). One reasonable way to boost native dextran production in *L. hordei* TMW 1.1822 may thus be to optimize constitutive dextransucrase accumulation. The cells could then be used as source of native dextransucrases that can be released by sucrose in minimal growth media without the aim of using the cells as growing metabolic machines.

As described for some other dextransucrases [16, 30, 40, 66, 68], maltose was more favored as acceptor than sucrose or its glucose residue. However, the elongation to isolable high molecular weight dextran was drastically inhibited (Fig. 3, bottom), leading to the assumption that the formed product panose was no suited acceptor for further glucose moieties. Due to the metabolization by various probiotic bacteria [69, 70], panose is regarded as prebiotic carbon source, whose production could be optimized by simple addition of maltose in addition to sucrose. However, this reaction could compete with the production of high molecular weight dextran in some fermented plant-based foods, which intrinsically contain maltose as degradation product of starch [71–73].

## Conclusion

This study reveals that the elongation activity of dextransucrases can be triggered by application of different production pH values or by addition of maltose. The properties of high molecular weight dextrans obtained at different pH values depend on the dextran concentration and especially on the molecular weight of the dextran molecules. These findings can be used for the controlled manufacture of functionally diverse products by the use of the dextransucrase of *L. hordei* TMW 1.1822 or other lactic acid bacteria.

## Data Availability

The datasets used and/or analyzed during the current study are available from the corresponding author on reasonable request.

## Abbreviations

AF4-MALS-UV: asymmetric flow field-flow fractionation coupled with multi-angle laser light scattering and UV detection
EPS: exopolysaccharide
fru: fructose
glc: glucose
HPAEC-PAD: high-performance anion-exchange chromatography with pulsed amperometric detection
HPLC-RI: high-performance liquid chromatography with refractive index detection
L.: *Liquorilactobacillus*
mal: maltose
MALDI-TOF-MS: matrix-assisted laser desorption–ionization time-of-flight mass spectrometry
rms: root mean square
suc: sucrose
TMW: Technische Mikrobiologie Weihenstephan
$$ \dot{\gamma} $$: shear rate
*η*: viscosity
G′: storage modulus
G″: loss modulus
*ω*: angular frequency
